# Left Ventricular Global Longitudinal Strain as a Parameter of Mild Myocardial Dysfunction in Athletes after COVID-19

**DOI:** 10.3390/jcdd10050189

**Published:** 2023-04-23

**Authors:** Jana Schellenberg, Magdalena Ahathaller, Lynn Matits, Johannes Kirsten, Johannes Kersten, Juergen Michael Steinacker

**Affiliations:** 1 Division of Sports and Rehabilitation Medicine, University Ulm Hospital, 89075 Ulm, Germany; magdalena.ahathaller@outlook.com (M.A.); lynn.matits@uni-ulm.de (L.M.); johannes.kirsten@uniklinik-ulm.de (J.K.); johannes.kersten@uniklinik-ulm.de (J.K.); juergen.steinacker@uniklinik-ulm.de (J.M.S.); 2 Clinical & Biological Psychology, Institute of Psychology and Education, Ulm University, 89075 Ulm, Germany

**Keywords:** sport, COVID-19, speckle-tracking echocardiography, myocardial dysfunction, exercise performance

## Abstract

Whether symptoms during COVID-19 contribute to impaired left ventricular (LV) function remains unclear. We determine LV global longitudinal strain (GLS) between athletes with a positive COVID-19 test (PCAt) and healthy control athletes (CON) and relate it to symptoms during COVID-19. GLS is determined in four-, two-, and three-chamber views and assessed offline by a blinded investigator in 88 PCAt (35% women) (training at least three times per week/>20 MET) and 52 CONs from the national or state squad (38% women) at a median of two months after COVID-19. The results show that the GLS is significantly lower (GLS −18.53 ± 1.94% vs. −19.94 ± 1.42%, *p* < 0.001) and diastolic function significantly reduces (E/A 1.54 ± 0.52 vs. 1.66 ± 0.43, *p* = 0.020; E/E′l 5.74 ± 1.74 vs. 5.22 ± 1.36, *p* = 0.024) in PCAt. There is no association between GLS and symptoms like resting or exertional dyspnea, palpitations, chest pain or increased resting heart rate. However, there is a trend toward a lower GLS in PCAt with subjectively perceived performance limitation (*p* = 0.054). A significantly lower GLS and diastolic function in PCAt compared with healthy peers may indicate mild myocardial dysfunction after COVID-19. However, the changes are within the normal range, so that clinical relevance is questionable. Further studies on the effect of lower GLS on performance parameters are necessary.

## 1. Introduction

Coronavirus Disease 2019 (COVID-19) is a systemic viral infection caused by Severe Acute Respiratory Syndrome-Coronavirus-2 (SARS-CoV-2) that primarily affects the respiratory system but can also cause myocardial damage [1,2,3]. Even supposedly healthy individuals and athletes may be limited by COVID-19 despite a normally good state of health and fitness. Studies of elite athletes have shown that the infection is often mild (46–82%) or asymptomatic (16–58%) [4,5,6,7]. The most commonly reported symptoms are fever, headache, limb and muscle pain, flu symptoms, fatigue and dyspnea [4,8,9]. In rare cases (1–3%), myocarditis may occur [10,11]. Most athletes can return to competitive and amateur sports after a training break adapted to the existing symptoms and, if necessary, a return-to-sport examination [12,13,14]. However, having passed through COVID-19 does not necessarily imply a complete recovery to the original health or performance status. The symptoms may persist for weeks and months after symptomatic as well as asymptomatic disease progression or reappear with latency [15]. Approximately 20–30% of SARS-CoV-2 positive patients in the normal population [16,17] and probably fewer athletes (1.2–40%) still have symptoms after acute infection [18,19]. Fatigue, neurocognitive impairment, exertional dyspnea (20–30%), exertional and non-exertional chest pain or palpitations (16%) may arise or persist after COVID-19 [13,17,20]. Acute as well as persistent symptoms can lead to athlete performance loss and even career termination if athletes are unable to return to their pre-COVID-19 performance. In this context, impaired left ventricular (LV) function could contribute to persistent symptoms and reduced performance. However, larger multicenter studies of return to sport in competitive athletes suggest cardiac involvement in only a few cases [4,10,11,21]. If cardiac symptoms exist during and/or after infection, a return-to-sport examination with echocardiography should be performed [13]. Here, LV function can be assessed with speckle-tracking echocardiography (STE) in addition to conventional echocardiographic indices [12]. Individual studies demonstrate reduced LV global longitudinal strain (GLS) with preserved Ejection Fraction (pEF) in the setting of acute SARS-CoV-2 infection in hospitalized patients regardless of infection severity [22,23,24,25] and in patients recovered from COVID-19 [26,27,28,29]. There are limited data on changes in LV GLS in athletes after COVID-19. In two studies, GLS is not altered after COVID-19 [30,31]. The aim of this prospective study is, first, to determine differences in LV GLS between athletes who have no history of LV dysfunction but have a positive COVID-19 test (PCAt) and healthy control athletes (CON). Second, we investigate whether there is an association between GLS and symptoms during COVID-19 to identify athletes in need of a more targeted follow-up.

## 2. Materials and Methods

### 2.1. Study Population

This prospective single-center cohort study includes 140 athletes presenting to the Ulm Clinic for Sports and Rehabilitation Medicine underwent transthoracic echocardiography between June 2020 and November 2021: 88 athletes with a history of a positive COVID-19 test (PCAt) at a median of 2.00 months (25% quantile = 1.00 months, 75% quantile = 5.00 months) after COVID-19 and 52 healthy athletes from the national or state squad as a control group (CON) presenting for annual pre-participation screenings. In both groups, there are athletes who participated in endurance sports, strength sports, team sports or technical sports. A consecutive inclusion of all study participants was performed. The inclusion criteria for both groups are: ≥18 years of age, training at least 3 times per week with more than 20 metabolic equivalents of task (MET) per week and for PCAt a positive SARS-CoV-2 PCR test or antibody detection with additional typical symptoms. The exclusion criteria for both groups are: acute or chronic medical conditions that precluded the planned physical examination, acute SARS-CoV-2 infection, refusal of peripheral venous blood sampling, inadequate German language skills and withdrawal from study participation. Fifty-seven PCAt (65%) responded to questionnaires about symptoms and complaints during COVID-19, which we include in our analysis. Participants provided written informed consent after being instructed of the study procedures. The study was conducted in accordance with the Declaration of Helsinki and approved by the local ethics committee of the University of Ulm (EK 408/20).

### 2.2. Echocardiography

An EPIQ 7 ultrasound system with a phased-array probe X5-1 (Philips GmbH, Hamburg, Germany) was to perform 140 echocardiographic examinations. The GLS and global radial strain (GRS) were determined in apical four-, two- and three-chamber views in the apical, midline, and basal segments [32]. They were determined offline using TomTec postprocessing software (2D Cardiac Performance Analysis, TomTec Imaging Systems, Unterschleissheim, Germany) by an investigator who was blinded to the group assignments. The endocardial contour was manually adjusted. A selection of 15 images was reviewed a second time by the same investigator and another time by a second blinded investigator to determine intrarater and interrater reliability. The following parameters were collected: end-diastolic volume (EDV), end-systolic volume (ESV), left ventricular mass, left ventricular Ejection Fraction (LV-EF by biplane LV planimetry by Simpson), fractional shortening (FS), GLS, GRS, stroke volume (SV) and resting heart rate (HR). Diastolic function was characterized by E/A ratio, E/E′lateral ratio, E/E′medial ratio, V_max_E, V_max_A and deceleration time (Dec Time) [33,34].

### 2.3. Statistical Analysis

Statistical analyses were performed using R version 4.1.1 [35]. The descriptive data are presented as mean (M) ± standard deviation (SD) or as median and 25% quantile and 75% quantile. Group differences were examined using unpaired-Wilcoxon-Tests. Correlations between GLS and age and BMI were analyzed using the Pearson-correlation coefficient (r) and Spearman’s ρ. Additional analyses comparing the frequency of clinically abnormal GLS values in PCAt and CON were calculated using the phi coefficient. To control for possible confounding variables (BMI, age, sex, HR, blood pressure and sports type), multivariate linear regression models were performed for the confounding variables separately. A *p*-value of <0.050 is considered significant.

## 3. Results

### 3.1. Cohort Characteristics

A total of 88 PCAts and 52 CONs are included in the statistical analysis. The groups do not differ in terms of sex, weight, height, systolic blood pressure or HR. A significant age difference is found between the PCAt and CON. The BMI and diastolic blood pressure are significantly different between the two groups (Table 1).

The differences in GLS persist even when age, HR, sex or BMI are included as control variables, i.e., the differences between PCAt and CON re not due to the difference in age or BMI between the groups (Table S1).

### 3.2. Echocardiographic Parameters

**Figure 1:**
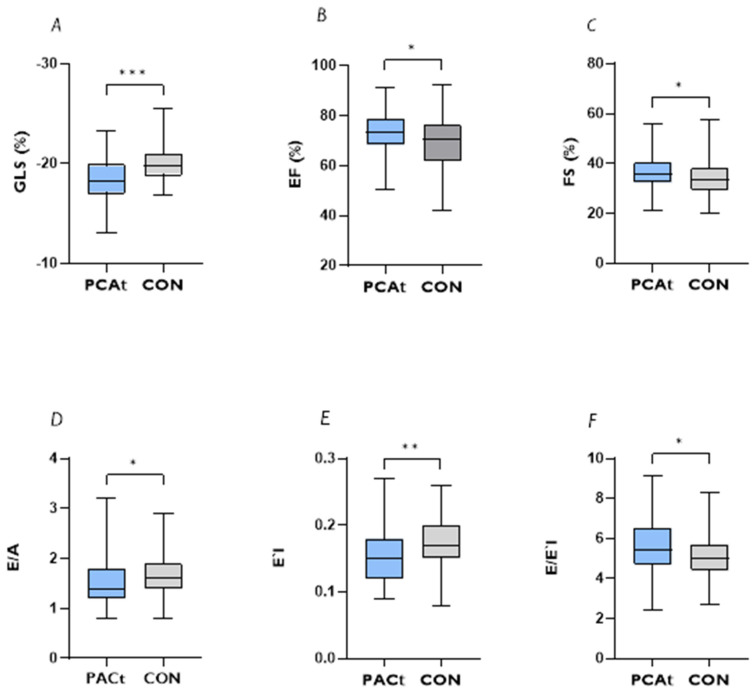
Differences in left ventricular parameters and diastolic function between athletes after COVID−19 (PCAt) and healthy federal squad athletes (CON). (**A**): Global longitudinal strain (GLS); (**B**): Ejection Fraction (EF); (**C**): Fractional shortening (FS); (**D**): E/A ratio (**E**): E′l; and (**F**): E/E′l. Significant results are presented as follows: * < 0.05 ** < 0.01 *** < 0.001. The results of the comparison between the PCAt and CON remain when age, HR, sex or BMI are included as control variables.

The absolute values of the echocardiographic parameters are given in Table 2. The PCAt shows significantly lower GLS values than the CON (Figure 1A). In both groups, GLS does not differ between men and women. Positive associations are found between GLS and BMI (ρ = 0.171, *p* = 0.025) in the total cohort. The Intrarater and interrater reliability with respect to the GLS measurement shows high agreement (intrarater: 0.892 [95%CI, 0.593–0.973]; interrater: 0.794 [95%CI, 0.159–0.949]. There is no significant association between GLS and age (ρ = 0.127, *p* = 0.093), GLS and the time period between examination and infection (ρ = 0.155, *p* = 0.115), GLS and HR (ρ = 0.146, *p* = 0.054) or between GLS and systolic (ρ = −0.110, *p* = 0.1646) and diastolic blood pressure (ρ = −0.06, *p* = 0.483). The LV-EF and FS are normal in both groups but significantly higher in the PCAt (Figure 1B,C). In the PCAt, the end-systolic volume is significantly smaller and the LV mass is significantly reduced (Table 2). The measured values of the diastolic function are within the normal range but there is a significant difference for E/A, E′lateral and E/E′lateral (Figure 1D–F). The extent of the GLS reduction is closely related to the E/A-Ratio (*p* < 0.001) and to V_max_E (*p* < 0.001). There re no significant differences in EDV, stroke volume and GRS between the PCAt and CON (Table 2). The differences in GLS persist when controlled for sex, age, BMI, systolic and diastolic blood pressure, heart rate and sports type (endurance sports, strength sports, team sports and technical sports) (Table S1).

### 3.3. Initial Symptoms and Correlation Analysis with GLS and GRS

The symptoms reported during the COVID-19 infection are classic symptoms of a viral infection: cough (55%), rhinitis (66%), exertional dyspnea (57%) and subjectively perceived reduction in performance (66%) compared with maximal performance before COVID-19. Even after infection, exertional dyspnea persisted in 62% (Table 3). The GLS and GRS values do not differ between the PCAt who reported symptoms during COVID-19 compared with asymptomatic PCAt (Tables S2 and S3). However, there is a trend toward lower GLS in PCAt with subjectively perceived performance limitations, but it is not significant (W = 417.0, *p* = 0.057).

## 4. Discussion

Cardiopulmonary symptoms after COVID-19 have been described, but not the influence of disease symptoms on cardiac function. In this prospective, single-center cohort study, we observe significantly lower GLS values and diastolic function in the PCAt. However, the changes are small and the measured values are within the normal range. Because this is a highly heterogeneous group of athletes with overlapping echocardiographic parameters, we hypothesize that the GLS differences may indicate mild myocardial dysfunction. The symptomatic athletes do not have different GLS or GRS values than asymptomatic athletes.

Acute SARS-CoV-2 infection in hospitalized patients shows a reduced GLS with pEF, regardless of the severity of infection [22,23,24], but the long-term consequences, e.g., in terms of mortality, incidence of heart failure or eventual recovery, are still unknown. A lower GLS has also been observed in patients with heart failure and pEF [36]. There is limited research on GLS in athletes after COVID-19. Fikenzer et al., find no echocardiographic GLS differences between eight infected and four uninfected elite handball players, but magnetic resonance imaging shows mild signs of acute inflammation/oedema in all infected athletes [31]. However, the study is very small and limited to male athletes. Data from the current literature describe a very low rate (1–3%) of myocarditis after COVID-19 [10,11]. Echocardiographic GLS measurement is a commonly available test and may be useful as an additional parameter in selecting athletes with an appropriate clinic for limited capacity MRI examinations [12]. Another study shows no COVID-19-mediated GLS differences in 107 elite athletes (23% women) with mild symptoms and 107 randomized healthy athletes [30]. However, in a subset of post-COVID athletes with early diastolic septal flattening, there is a relative decrease in GLS in the free wall segments, suggesting a characteristic feature of pericardial constriction. Concerning the LV-EF, however, our results are in line with Lakatos et al. showing increased LV-EF in the PCAt compared with CON. An increased LV-EF can result from a reduced training volume, since LV-EF is often low, normal [37] or even slightly reduced in athletes [30,38]. Here, longitudinal investigations of GLS and LV-EF in detraining would be necessary.

Decreased GLS as an early marker of myocardial dysfunction could potentially contribute to persistent cardiopulmonary symptoms and subjectively perceived performance limitation after COVID-19. We investigate whether symptomatic courses lead to cardiac sequelae but find no differences in the GLS or GRS between symptomatic and asymptomatic athletes. Classic symptoms such as cough, rhinitis, sore throat, exertional dyspnea and subjectively perceived performance limitations are present in more than half of the PCAt, as reported in several studies [4,8,9]. Even after infection, exertional dyspnea remains in 62% of the PCAt, which is significantly more frequent than 20–30% reported in the literature [13,20]. Reasons for this could be that first, athletes might notice small changes in form of exertional dyspnea earlier and more than non-athletes, or secondly, it could be that mainly athletes with complaints presented themselves to our department. However, there is a trend toward a lower GLS in the PCAt with subjectively perceived performance limitation. A direct effect of reduced GLS on performance does not yet exist and should be evaluated in further studies. To our knowledge, this is the first study to evaluate individual symptoms rather than the severity of course. Post-COVID patients with significant functional impairment are more likely to have impaired GLS or cardiovascular comorbidities whereas those with mild to moderate functional impairment have no cardiovascular changes [26]. Similar results have been obtained by Mahajan et al., who describe an increasing impairment of GLS in mild (13.1%), moderate (44%) and severe (90%) disease [27]. These patients are older than our cohort of athletes and have concomitant cardiovascular diseases such as hypertension and diabetes mellitus. Furthermore, they are examined earlier (30–45 days after infection) than ours, so improvement of LV dysfunction cannot be excluded. However, our results argue against a temporal component: There was no significant association between the GLS and the time period between examination and infection, which could indicate a possible stable long-term decrease in the GLS in the PCAt. More time and a longer follow-up could be required for demonstrating a complete recovery (Long-COVID syndrome). A longer follow-up and longitudinal data should be required.

Normal ranges for the GLS are known to vary depending on common covariates such as age, sex, BMI or blood pressure [39,40], and also to vary between vendors [41]. In our study, there is a significant age difference between the PCAt and CON, but no positive correlation between GLS and age in the overall cohort. Zghal et al. find a decrease in GLS with age with no change in LV-EF [42]. In the Danish City of Copenhagen Heart Study GLS changes differently with age in men and women, with men having lower mean values and lower reference limits for all exercise parameters. 62% of the participants are female and older with an age of 46 ± 16 years compared with our study, which has 45% women and ages of 31.44 ± 12.62 (PCAt) and 24.69 ± 7.89 (CON) years, respectively [43]. In our study, GLS does not differ between men and women in both the PCAt and CON groups. Although the diastolic blood pressure is significantly different between the two groups, there was no association between GLS and systolic or diastolic blood pressure. Blood pressure is known to correlate with GLS [44,45], and it has been shown that GLS is significantly reduced in patients with hypertension compared with normotensive control subjects [46]. Due to the specific athlete clientele, the variance of the blood pressure values is low, which is accompanied by a limited interpretability of the correlation. In addition, the test used (Wilcoxon) compares rank sums and not medians or means. In our study, there is a positive association between the GLS and BMI. Impairment of the LV-EF and GLS by overweight and obesity is also known from other studies [47,48,49]. The parameters of diastolic function are within the normal range in our studied athletes but are significantly reduced in the PCAt. Some authors indicate that diastolic function is normal or decreased in athletes [50,51] but may also be supranormal in trained endurance athletes compared with untrained individuals [52]. According to the study by Galderisi et al., the extent of GLS is closely related to diastolic function [51]. We also demonstrate a correlation of the GLS with the E/A ratio and V_max_E. Thus, a routine determination of diastolic function may be an initial clue to possible LV dysfunction and should be followed by determination of the GLS at the latest in case of abnormalities.

### Strengths and Limitations

This study is limited by the cross-sectional design, as no prior strain values of the PCAt and CON are available. Therefore, it cannot be excluded that reduced strain values did exist preliminarily or are due to sport-related adjustments. Because of the nature of this study, the time after the SARS-CoV-2 infection differed between subjects, and heart function may have already improved or worsened during infection, perhaps as a result of detraining. However, there is no significant correlation between the GLS and the time between examination and infection. Because only 65% of the PCATs completed the questionnaires about symptoms and complaints during COVID-19, we are unable to include all of the PCATs in the analysis, which may have influenced our results. The clearly defined population of study participants, consisting of athletes who did not differ significantly clinically, limits the generalizability of the results for the general population, but on the other hand, allows an assessment of a specific group. The difference in training volume between and within groups could affect our results. However, even when the groups differ significantly in age and diastolic blood pressure, no significant association between GLS and age and GLS and diastolic blood pressure is found. The differences in GLS persist even when sex, age, BMI, systolic and diastolic blood pressure, heart rate and sports type are included as control variables, i.e., the differences between the PCAt and CON are not due to the difference in age or BMI between the groups. Although the GLS determination can be software and investigator experience dependent, our results show high intrarater and interrater reliability. The possible influence on myocardial strain parameters exerted by chest wall conformation is not specifically assessed in our cohort of athletes [53,54]. It should be emphasized that we achieve a meaningful case number of athletes for a single-center study. Finally, although an association between COVID-19 and the occurrence of pathological examination findings up to myocarditis is suggested, direct evidence is still lacking.

## 5. Conclusions

We observe significantly lower GLS values and diastolic function in PCAt compared with healthy peers. Because this is a highly heterogeneous group of athletes with overlapping echocardiographic parameters, we hypothesize that the GLS differences may indicate mild myocardial dysfunction. However, the changes are within the normal range, so that clinical relevance is questionable. Symptomatic athletes do not have different GLS or GRS values than asymptomatic athletes. In diagnostics, this does not result in fundamental changes, but when it comes to success and failure, medals or top rankings, small differences in performance can be decisive. Therefore, in addition to factors such as lack of training, myocardial damage could also affect success. Further studies on the effect of lower GLS on performance parameters would be informative, as would long-term observations in athletes and in the general population to evaluate the effects of COVID-19 on cardiac function and performance impairment and for demonstrating a possible complete recovery.

## Figures and Tables

**Table 1:** Cohort characteristics subdivided in athletes after COVID-19 (PCAt) and healthy athletes (CON).

	PCAt	CON	Test Statistic	*p*-Value
***Number***	88	52		
***Sex (female/male)***	31/57 (35%/65%)	20/32 (38%/62%)	φ = 0.04	0.741
***Age (years), median (IQR)***	29 (22–40)	22 (19–28)	W = 2655.5	<0.010 **
***Weight (kg), median (IQR)***	73.3 (64.2–84.5)	72.4 (61.4–80.9)	W = 2735	0.212
***Height (cm), median (IQR)***	176 (168–189)	179 (171–184)	W = 3332.5	0.4072
***BMI (kg/m^2^), median (IQR)***	22.8 (21.6–25.9)	22.7 (20.8–24.3)	W = 2522.5	<0.050 *
***Systolic blood pressure, mmHg, median (IQR)***	120 (110–125)	120 (110–130)	W = 2746	0.1418
***Diastolic blood pressure, mmHg, median (IQR)***	80 (70–80)	80 (71.25–90)	W = 2559	0.033 *
***HR (bpm), median (IQR)***	62 (55–68)	62 (56–68)	W = 5709	0.6317
***Training volume***	at least three times per week >20 MET	>three times per week >20 MET		

Abbreviations: W = (two-tailed unpaired) Wilcoxon Signed Rank Test or φ Phi-coefficient, IQR = interquartile range, BMI = body mass index, HR = heart rate bpm beats per minute and MET = metabolic equivalents of task. Significant results are presented as follows: * < 0.05 ** < 0.01.

**Table 2:** Echocardiographic parameters subdivided in athletes after COVID-19 (PCAt) and healthy athletes (CON).

	PCAt	CON	W	*p*-Value
***EDV (mL), median (IQR)***	123.00 (108.00–146.00)	141.50 (104.00–169.00)	4207.5	0.246
***ESV (mL), median (IQR)***	32.50 (24.18–44.53)	38.80 (28.62–53.40)	4488.0	0.047 *
***LV Mass (g), median (IQR)***	149.50 (124.00–184.75)	172.00 (134.00–202.50)	4457.5	0.037 *
***LV-EF (%), median (IQR)***	73.25 (67.85–78.57)	70.35 (61.95–76.00)	3130.5	0.042 *
***FS (%), median (IQR)***	35.75 (32.32–40.30)	33.40 (29.35–38.00)	2949.0	0.015 *
***GLS (%), median (IQR)***	−18.27 (−19.72–−17.14)	−19.76 (−20.72–18.99)	2059.5	<0.001 ***
***GRS (%), median (IQR)***	10.42 (5.60–15.91)	11.12 (6.11–19.45)	4020.5	0.349
***Stroke Volume (ml), median (IQR)***	89.10 (77.60–110.00)	92.30 (71.85–115.00)	3788.5	0.855
***E/A, median (IQR)***	1.40 (1.20–1.80)	1.60 (1.40–1.90)	4416.0	0.020 *
***E′l, median (IQR)***	0.15 (0.12–0.18)	0.16 (0.15–0.20)	4057.0	0.009 **
***E′m, median (IQR)***	0.11 (0.09–0.13)	0.12 (0.10–0.13)	3129.5	0.414
***E/E′l, median (IQR)***	5.45 (4.70–6.60)	5.00 (4.40–5.70)	2629.0	0.024 *
***E/E′m, median (IQR)***	7.30 (6.30–9.15)	7.20 (6.50–8.10)	3585.5	0.618
***V_max_ A, median (IQR)***	0.55 (0.46–0.64)	0.52 (0.46–0.58)	3078.0	0.076
***V_max_ E, median (IQR)***	0.81 (0.67–0.96)	0.83 (0.72–0.94)	3974.0	0.332
***Dec Time, median (IQR)***	164.00 (129.00–198.00)	183.50 (144.25–218.50)	2415.0	0.168

Abbreviations: W = (two-tailed unpaired) Wilcoxon Signed Rank Test, IQR = interquartile range, EDV = end-diastolic volume, ESV = end-systolic volume, LV mass = left ventricular mass, LV-EF = left ventricular ejection fraction by Simpson, FS = fractional shortening, GLS = global longitudinal strain, GRS = global radial strain, E/A ratio, E/E′l ratio E/E′m ratio, V_max_ A = velocity of A wave, V_max_ E = velocity of E wave, Dec Time=Deceleration Time. Significant results are presented as follows: * < 0.05 ** < 0.01 *** < 0.001.

**Table 3:** Symptoms during COVID-19 in athletes after COVID-19 (PCAt) presented as absolute values and relative frequencies.

Symptoms	Present	Not Present
fever	26 (46%)	30 (54%)
cough	31 (55%)	25 (45%)
rhinorrhea	37 (66%)	19 (34%)
sore throat	33 (59%)	23 (41%)
resting dyspnea	14 (25%)	42 (75%)
exertional dyspnea during COVID-19	32 (57%)	24 (43%)
exertional dyspnea after COVID-19	34 (62%)	21 (38%)
palpitations	20 (36%)	36 (64%)
chest pain	20 (36%)	36 (64%)
increased resting heart rate	25 (45%)	31 (55%)
subjective perceived performance limitation	37 (66%)	19 (34%)
dizziness	25 (45%)	30 (55%)

## Data Availability

The data underlying this article will be shared on reasonable request to the corresponding author.

## Supplementary Materials

The following supporting information can be downloaded at: https://www.mdpi.com/article/10.3390/jcdd10050189/s1, Table S1: Multivariate linear regression for confounding variables, Table S2: Symptoms during COVID-19 in correlation with GLS presented as median and IQR, Table S3: Symptoms during COVID-19 in correlation with GRS presented as median and IQR.
