# Supplementary material for: Left Ventricular Global Longitudinal Strain as a Parameter of Mild Myocardial Dysfunction in Athletes after COVID-19
Source: J Cardiovasc Dev Dis. 2023 Apr 23;10(5):189. doi: 10.3390/jcdd10050189 (PMC10218982; doi:10.3390/jcdd10050189)
Supplement: Supplementary file 1 [file jcdd-10-00189-s001.zip › jcdd-2343870-supplementary.pdf]

Supplementary Data:

Table S1. Multivariate linear Regression for confounding variables.

Parameter	Coefficient	SE	95% CI	t	p
(Intercept)	−19.72	0.23	[−20.17, −19.27]	−86.58	< 0.001
Group	−1.36	0.27	[0.84, 1.89]	5.14	< 0.001
Sex	−0.42	0.27	[−0.95, 0.12]	−1.53	0.127
(Intercept)	−20.12	0.36	[−20.84, −19.40]	−55.21	< 0.001
Group	−1.33	0.28	[0.78, 1.89]	4.71	< 0.001
Age	0.009	0.01	[−0.01, 0.03]	0.81	0.420
(Intercept)	−21.42	1.01	[−23.42, −19.43]	−21.20	< .001
Group	−1.44	0.30	[0.85, 2.02]	4.86	< 0.001
BMI	0.07	0.04	[−0.02, 0.15]	−1.51	0.133
(Intercept)	−18.77	1.23	[−21.19, −16.35]	−15.29	< 0.001
Time	1.42	0.26	[0.90, 1.94]	5.42	< 0.001
Systolic BP	0.009	0.009	[−0.03, 0.01]	−0.95	0.346
(Intercept)	−19.67	1.05	[−21.73, −17.60]	−18.82	< 0.001
Group	1.44	0.27	[0.91, 1.98]	5.33	< 0.001
Diastolic BP	0.003	0.01	[−0.03, 0.02]	−0.25	0.801
(Intercept)	−21.66	0.85	[−23.34, −19.98]	−25.43	< 0.001
Group	1.39	0.27	[0.86, 1.92]	5.20	< 0.001
HR	0.03	0.01	[0.00, 0.05]	2.17	0.032
(Intercept)	−19.64	0.26	[−20.15, −19.14]	−76.90	< 0.001
Group	1.38	0.27	[0.87, 1.98]	5.16	< 0.001
Endurance sports	−0.40	0.26	[−1.09, 0.79]	−1.54	0.126
(Intercept)	−19.91	0.23	[−20.36, −19.46]	−87.69	< 0.001
Group	1.42	0.28	[0.88, 1.97]	5.12	< 0.001
Strength sports	0.07	0.38	[−0.68, 0.83]	0.19	0.848
(Intercept)	−19.94	0.21	[−20.35, −19.52]	−95.63	< .001
Group	1.23	0.28	[0.69, 1.78]	4.45	< .001
Team sports	0.61	0.33	[−0.03, 1.25]	1.88	0.062
(Intercept)	−19.90	0.23	[−20.35, −19.45]	−86.79	< 0.001
Group	1.43	0.28	[0.87, 1.98]	5.06	< 0.001
Technical sports	−0.15	0.48	[−1.09, 0.79]	−0.31	0.760

Abbreviations: SE standard error, CI confidence interval, BMI body mass index, BP blood pressure HR heart rate.

Table S2. Symptoms during COVID-19 in correlation with GLS presented as median and IQR.

	GLS			
	Present Symptoms	Not present Symptoms	W	p-Value
fever	−17.73 [−19.71, −16.90]	−17.93 [−19.21, −17.01]	372.5	0.890
cough	−17.35 [−19.43, −16.97]	−18.17 [−19.32, −16.90]	304.0	0.363
rhinitis	−17.83 [−19.58, −17.06]	−17.83 [19.25, −16.90]	352.0	0.731
sore throat	−17.38 [−19.05, −16.53]	−18.11 [−20.22, −17.23]	232.5	0.036
resting dyspnea	−17.93 [19.59, −16.99]	−17.78 [−19.30, −16.91]	302.0	0.671
exertional dyspnea during COVID-19	−17.73 [−19.57, −17.09]	−18.05 [−19.31, −16.76]	357.0	0.937
exertional dyspnea after COVID-19	−17.83 [−19.59, −17.16]	−18.04 [−19.30, −16.45]	359.0	0.510
palpitations	−18.05 –[19.29, −17.30]	−17.58 [−19.33, −16.76]	385.0	0.435
chest pain	−17.63 [−19.77, −16.71]	−17.93 [−19.27, −16.95]	340.5	1.000
increased resting heart rate	−17.93 [−19.64, −16.78]	−17.78 [−19.28, −16.95]	361.5	0.986
subjective perceived performance limitation	−17.05 [−18.05, −16.46]	−18.03 [−19.60, −17.19]	417.0	0.057
dizziness	−17.83 [−19.76, −17.05]	−17.83 [−19.29, −16.89]	394.5	0.585

Abbreviations: IQR interquartile range W (two-tailed unpaired) Wilcoxon Signed Rank Test.

Table S3. Symptoms during COVID-19 in correlation with GRS presented as median and IQR.

	GRS			
	**Present Symptoms**	**Not present Symptoms**	**W**	**p-value**
fever	8.53 [4.33, 14.60]	6.31 [0.86, 14.83]	329.0	0.553
cough	6.27 [2.88, 15.40]	8.16 [5.69, 14.04]	382.0	0.665
rhinitis	6.78 [3.45, 15.40]	6.49 [1.67, 12.18]	304.0	0.615
sore throat	6.38 [3.67, 14.90]	7.74 [3.26, 14.53]	375.0	0.695
resting dyspnea	12.04 [6.27, 17.24]	6.32 [0.86, 14.02]	195.0	0.096
exertional dyspnea during COVID-19	6.64 [2.91, 14.093]	6.84 [4.01, 14.50]	355.0	0.965
exertional dyspnea after COVID-19	7.47 [4.22, 14.60]	6.27 [−0.42, 12.94]	287.0	0.514
palpitations	8.06 [5.01, 15.54]	6.43 [−0.69, 14.12]	279.0	0.281
chest pain	5.84 [2.80, 15.60]	8.75 [3.19, 14.13]	368.0	0.625
increased resting heart rate	6.29 [3.95, 15.54]	7.05 [3.10, 14.13]	364.0	0.952
subjective perceived performance limitation	6.36 [0.11, 14.01]	6.78 [4.20, 15.47]	291.0	0.671
dizziness	6.19 [3.95, 14.75]	10.31 [1.11, 14.66]	394.0	0.594

Abbreviations: IQR interquartile range W (two-tailed unpaired) Wilcoxon Signed Rank Test.
